# New Medicine for the New Times

**Published:** 1892-11-12

**Authors:** 


### The Hospital

An Institutional Journal of

Science, Medicine, Nursing, and Philanthropy.

*For Hospitals, Asylums, Infirmaries, Dispensaries, Medical Practitioners, Students, Nurses, and the Charitable Public.*

Vol. XIII.—No. 320.] [Nov. 12, 1892.

### New Medicine for the New Times.

Men whose minds feed exclusively upon ideas; men who do not put forth their hands upon external and physically actual things, may and do move, but they are apt to move in a circle; they may make no progress towards a definite and desired goal. Medicine is moving; the whole world is conscious of its movement. Is the movement forward, or is it only a series of wearisome revolutions which always end exactly where they began? We shall hazard the opinion that medicine has moved faster and farther in a forward direction than any other combined science and art during the last half century.

The fate of the world's immediate future is bound up with that of scientific medicine. Physic is no longer a healing art pure and simple; it is the science of man. The only persons who have told the world anything new about man during the last three centuries have been the physiologists and the doctors. Before the advent of the modern physiologist man was a mystery, physically as well as psychologically. But the physical mystery has been penetrated from a thousand sides, and man now stands revealed to the physiologist as in the broad light of noonday. The physiologist knows man to-day, the practising doctor will know him to-morrow, and the great world will succeed to this revolutionising knowledge in good time. The future of the world lies with the men who are imbued with the knowledge of physical science, and who have the power of rendering effectual service in the struggles and conflicts which loom in the near to-morrow.

A new conception of the physician is forming itself in men's minds. He is not now the mere guesser who has certain selected remedies in his armamentarium which he applies in faith and hope to ills of which he only half understands the nature. The distinguishing characteristic of the new medicine is its clear knowledge. The skilful modern physician can, so to speak, analyse and synthesise his patient with as much exactitude as the chemist can break down his marble and extract the desired acid, or reconstruct his calcium carbonate by breathing into lime water. To the competent diagnostician the sick patient is an open book, a book of which every paragraph and line can be distinctly read and the meaning thereof scientifically interpreted. It is a great thing to be able to examine any subject from the inside. That is what the modern physician practically does in his daily work. The discovery made by Dr. Rich .rdson the other day, whereby with the aid of the stomach tube and the stethoscope an approach can be made to such close contact, not only with the stomach but with the heart and lungs, as almost to amount to an immediate aural inspection of those organs, is exactly on the lines of all previous advances in the diagnostician's art. Dr. Richardson is a veteran at the manifold arts of physical investigation, and it was peculiarly appropriate that to him should be revealed, not by accident, but by the persevering application of well established principles, a more intimate manifestation of those vital organs in their actual working than has ever been witnessed before.

There is, however, one serious circumstance associated with modern medicine which it behoves all loyal men of science immediately to take into their consideration; and that is the general disjointedness and want of concentration which the art and its practitioners, as a whole, display. The thirty thousand medical men of this country ought to be a conquering army, subjugating all the material ignorance and darkness of their time, and guiding the millions of our people into the ways of intelligent physical well being, and victorious material and moral progress. But for practical purposes, scientific doctors are not an army but a mob; and too often a paralysed mob. The disorganisations of medicine is absolute and complete. The medical profession in this country consists of thirty thousand separate men of business, every one of them fighting for his own hand.

Now, here is a proposition which no man who thinks for a single moment will venture to deny: the thirty thousand doctors of our country exist, in ultimate analysis, for the protection of the health, happiness, and general well-being of the whole community. They do not exist for the low and poor purpose of administering a pill to this person who feels out of sorts, or a potion to that one who thinks he would like a little medicine. The medical science of to-day has far higher objects; objects that demand a transference of the right of initiative. As things stand at present the ignorant patient decides for himself when and where he will consult the physician: and he often delays the decision so long that it is too late to consult him at all. But in a highly-complex and danger-charged environment like that of the civilised world of to-day, such an arrangement displays the very maximum of ineptitude and folly. It is as if the pupils at a school, and not the master, should fix their own lessons and the hours of their attendance in class. It is easy to imagine what proportion work would bear to play under such an arrangement, and also what sort of finished products such schoolboys would certainly be.

There would be almost insuperable difficulties, argues the timid man, in the way of transferring the initiative from the patient to the doctor on anything like an universal scale. Most true; but it is one of the firmest of all our convictions that the difficulties of life are increasing at a constantly accelerating pace, and that the man or the nation which feels no competency for dealing with difficulties and novel situations is destined very soon to join the "submerged tenth."

There are directly in front of the people of this country, practical problems of such a kind as the world has never yet successfully solved. One of the most urgent of all these is the problem of keeping ourselves alive and in fair working health. For if any considerable number of our people lose their power of work, and of good work, it is manifest that our national and individual days will be speedily numbered. Now it is clear that our thirty thousand medical men, if organised and constituted into an army of teachers, helpers, and trusted directors for each and every part of the community, could do infinitely more for the general and individual well-being than it is possible for them to do when every one of them fights for his own hand. If any one doubts this let him think, first, of the effect of sending a well-drilled and well-officered army of thirty thousand men into an enemy's country; and, secondly, of sending into the same country thirty thousand separate individuals, without officers, without objective, and everyone seeking his own pleasure and advantage. The first thirty thousand, being a controlled and directed army, might conquer the country and reconstruct its civilisation. The second thirty thousand would succeed in effecting nothing at all, but would everyone be cut to pieces in detail.

It is our contention that the splendid accumulations of scientific knowledge which have been gained by the medical profession during the last half century, are, so far as the country at large is concerned, practically wasted. This is especially the case with medical knowledge. It is gold of the finest quality; but its use is so limited that it might almost as well be still in the mine. In Bacon's days the general knowledge bore some relationship to the state of knowledge in the medical profession. At present the general public is living medically in the darkness of midnight, whilst the average doctor works in broad daylight. This is fatal to the world. The medical leadership which Mr. Gladstone predicts is the new way of safety for the difficulties and struggles of the new time. Doctors do not all see the new work to which destiny is calling them: and the people, unhappily, do not see their own need of the help and guidance which scientific medicine can give. That man who can so organise the new medicine as to make all its treasures of knowledge available for the whole people, will be one of the greatest benefactors of his time.

